# Supplementary material for: Spectral decoupling for training transferable neural networks in medical imaging
Source: iScience. 2022 Jan 17;25(2):103767. doi: 10.1016/j.isci.2022.103767 (PMC8816718; doi:10.1016/j.isci.2022.103767)
Supplement: Document S1. Figure S1 [file mmc1.pdf]

iScience, Volume *25*

Supplemental information

Spectral decoupling for training transferable neural networks in medical imaging

Joona Pohjonen, Carolin Stürenberg, Antti Rannikko, Tuomas Mirtti, and Esa Pitkänen

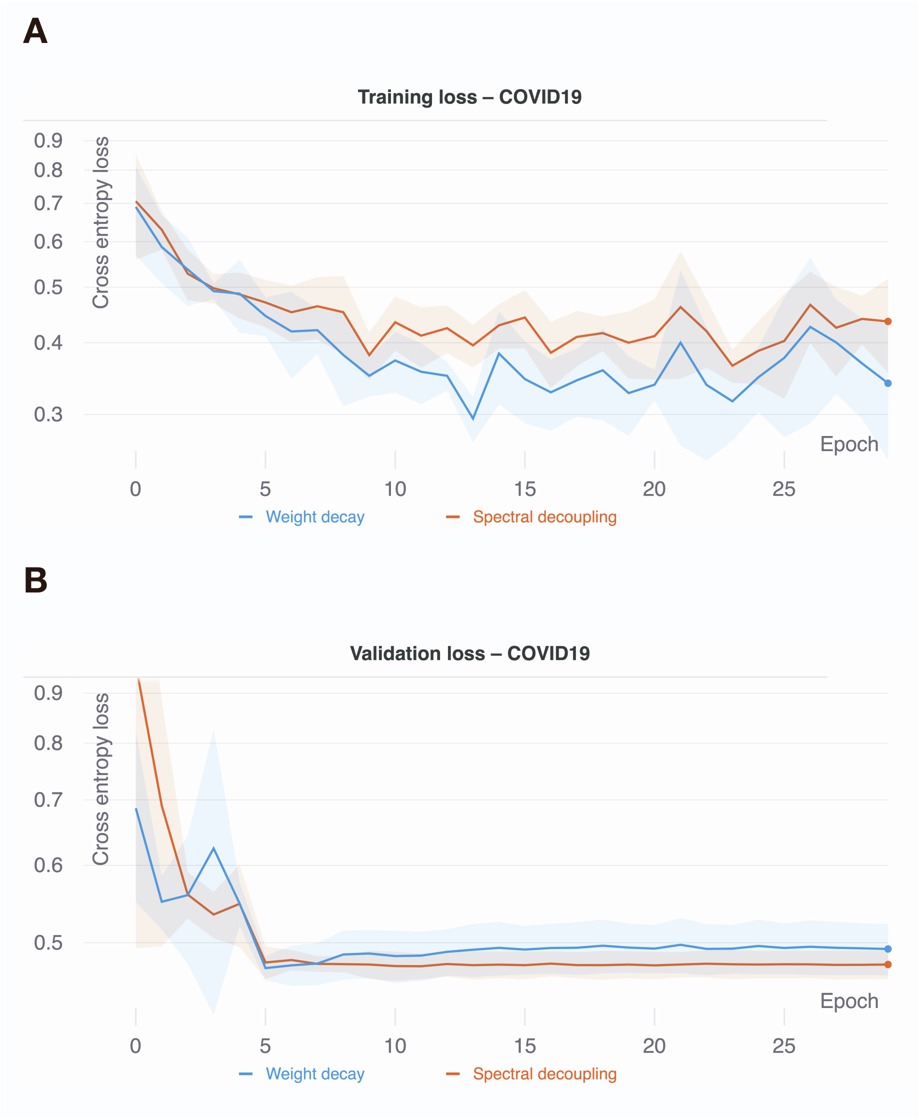

Figure S1: Examples of how training and validation curves were used in COVID-19 detection (Section 2.4). (**A**) Training loss curves for the neural networks trained for COVID-19 detection. Each training run trains similarly and converges to a similar training loss. Networks trained with spectral decoupling have a slightly higher training loss due to the added L2 norm. (**B**) Validation loss curves for the neural networks trained for COVID-19 detection. Each neural network converges to a similar loss and does not overfit. Networks trained with spectral decoupling achieve on average a lower validation loss.
