# Supplementary figures and images for: Spectral decoupling for training transferable neural networks in medical imaging
Source: iScience. 2022 Jan 17;25(2):103767. doi: 10.1016/j.isci.2022.103767 (PMC8816718; doi:10.1016/j.isci.2022.103767)

**A****Training loss – COVID19**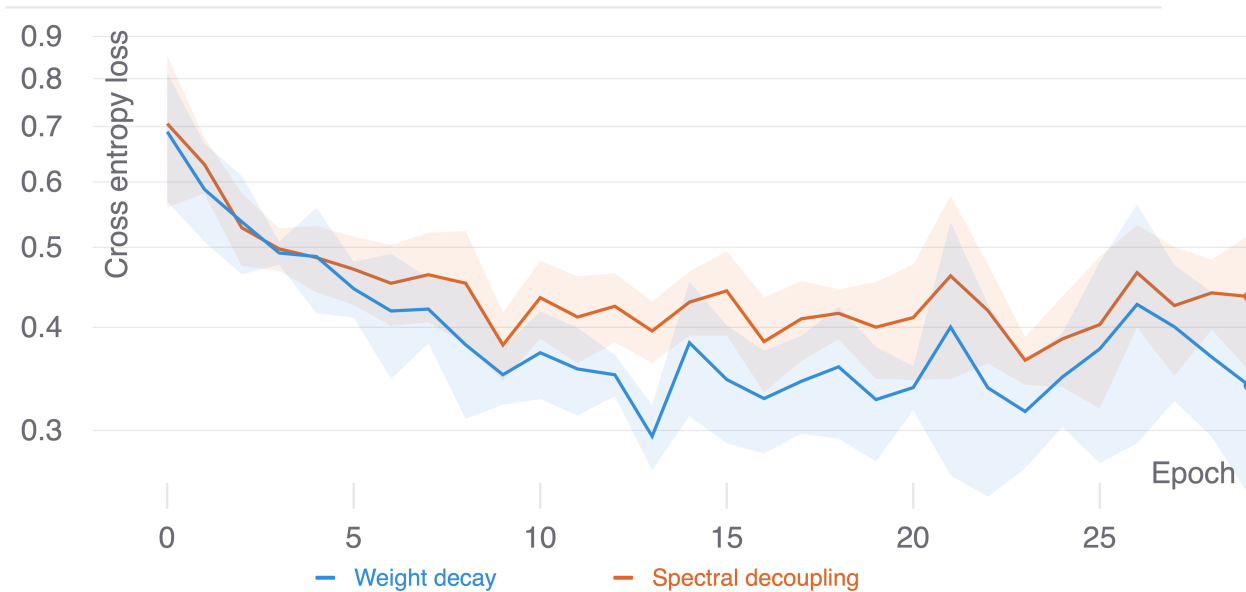**B****Validation loss – COVID19**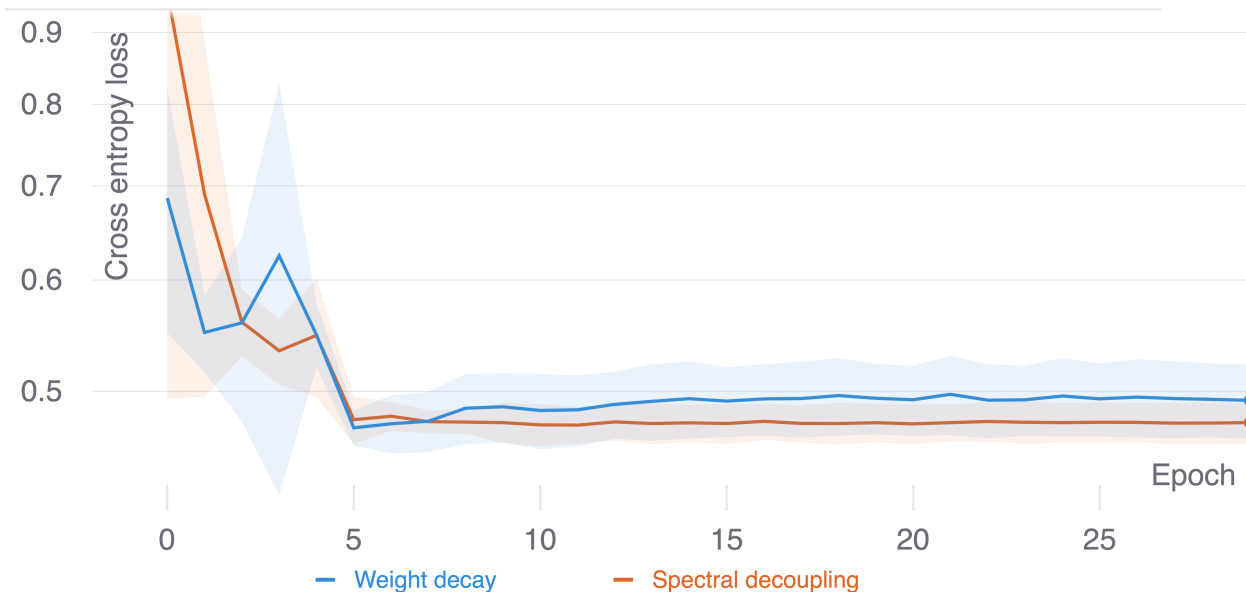

Supplement: Data S1. Training and validation curves for COVID-19 detection [file mmc2.pdf]
